# Genetic analyses of lumbosacral transitional vertebra and hip dysplasia in nine dog breeds in Norway

**DOI:** 10.1186/s13028-025-00810-z

**Published:** 2025-05-26

**Authors:** Jon Andre Berg, Bente Kristin Sævik, Cathrine Trangerud, Per Madsen, Frode Lingaas

**Affiliations:** 1 Department of Preclinical Sciences and Pathology, Faculty of Veterinary Medicine, Norwegian University of Life Sciences, Oluf Thesens Vei 30, Ås, 1432 Norway; 2 AniCura Jeløy Dyresykehus, Varnaveien 43d, Moss, 1526 Norway; 3 Department of Companion Animal Clinical Sciences, Faculty of Veterinary Medicine, Norwegian University of Life Sciences, Oluf Thesens Vei 30, Ås, Norway; 4 Veterinærradiologene AS, Skytta terrasse 2, Hagan, 1481 Norway; 5 Center for Quantitative Genetics and Genomics, Aarhus University, Aarhus, DK-8000 Denmark

**Keywords:** Canine, CHD, Heritability, Inheritance, LTV, Transitional vertebra

## Abstract

**Background:**

A lumbosacral transitional vertebra (LTV) is a congenital anomaly with reported prevalences ranging from 0 to 67% in different dog breeds, implying possible genetic differences. LTV has been associated with canine hip dysplasia (CHD) and degenerative lumbosacral stenosis (DLSS). Genetic parameters, including heritability estimates, are important for understanding the genetic influence on specific traits and for evaluating the effectiveness of possible genetic selection in reducing the prevalence of disorders. This study aimed to determine the heritability of LTV in nine dog breeds in Norway and estimate the genetic correlation with CHD.

**Results:**

The heritability estimates for LTV across the nine breeds ranged from low to moderate (0.056–0.314), while the heritability estimates for CHD were moderate to high (0.254–0.580). The estimates of genetic correlations between the two traits were mostly non-significant and varied strongly among breeds in size and direction.

**Conclusions:**

This study indicated that genetic factors influence LTV in several breeds and that there is a potential to reduce the prevalence by genetic selection, even if the heritability estimates of LTV ranged from low to moderate. The heritability estimates of CHD were within the range reported earlier, ranging from moderate to high. There was no general indication of a genetic correlation between LTV and CHD across breeds.

**Supplementary Information:**

The online version contains supplementary material available at 10.1186/s13028-025-00810-z.

## Background

A lumbosacral transitional vertebra (LTV) in dogs is a congenitally malformed vertebra located at the junction between the last normal lumbar vertebra and the sacrum [1]. The prevalence of LTV has been reported to vary by geographical location, classification system, and breeds included, ranging from 0 to 67% [1–8]. The significant difference in prevalences among dog breeds implies that genetic differences exist [2, 3, 7].

The heritability expresses how much of a trait’s phenotypic variance can be explained by additive genetic variance and ranges from 0 to 1. Heritability less than 0.2 is considered low; those with values between 0.2 and 0.5 are moderate, and those with values between 0.5 and 1.0 are highly heritable [9]. Generally, the higher the heritability, the stronger the influence of genetic effects on the trait and, thus, the greater the potential response to selective breeding [10].

The estimated heritability of canine hip dysplasia (CHD) has been reported to be > 0.20 in many breeds [11–15]. Systematic selection programs have resulted in significant genetic progress for many breeds and working dog populations [16, 17]. The heritability of LTV in German Shepherd dogs (GSD) has also been reported to be moderate to high [8, 18, 19], indicating a potential to reduce the prevalence through breeding [19]. However, limited information exists on the heritability of LTV in other breeds of dogs.

A recent study found that GSD with LTV type 1 have a higher risk of producing progeny with LTV types 2 and 3 compared to dogs with normal lumbosacral anatomy [18]. In that study, LTV was classified accordingly: LTV type 1 is characterised by an independent spinous process of the first sacral vertebra, which is separated from the medial sacral crest; LTV type 2 is a symmetrical form of a transitional vertebra, separated from the sacrum with an intervertebral space, and LTV type 3 is characterised by its asymmetrical morphology. LTV type 0 radiographically designates a dog with normal lumbosacral anatomy [20]. An eighth lumbar vertebra has been identified as a marker for LTV [6], with studies showing a high co-occurrence of this trait between parents and offspring. Research on the genetic basis of lumbosacral phenotypes suggests that both the presence of this vertebra and the fusion between the sacrum and first coccygeal vertebra should be included in LTV screening, as they likely share a common genetic origin [21].

Certain LTV types have been associated with CHD, asymmetric hip grades, and degenerative lumbosacral stenosis (DLSS) [2, 22–26]. Moreover, DLSS is a recognised cause for early retirement among service dogs in law enforcement and military roles [27, 28]. Additionally, CHD and DLSS are common causes of veterinary visits, where both diseases can lead to chronic pain, reduced quality of life, and altered behaviour [27–30]. Therefore, LTV may contribute to immediate and long-term discomfort and health issues, compromising overall animal welfare, with added emotional and financial burdens on owners [31, 32].

This study aimed to estimate the heritability (*h²*) for LTV and the additive genetic correlation between LTV and CHD in nine dog breeds in Norway. Identifying a positive correlation would suggest that breeding programs for CHD might also have a beneficial effect on the prevalences of LTV.

## Methods

### Data source

Breed selection was based on the evaluation of ventrodorsal (VD) radiographs from the Norwegian Kennel Club (NKK) database [33]. The database includes radiographs collected as part of the screening programme for CHD in Norway. Data from February 2014 to January 2022 were used, as this period ensured that radiographs were available in digital format, facilitating detailed and consistent classification of LTV [2] and enabling heritability estimation for LTV. More extensive historical data was available to estimate the heritability of CHD in the same dog breeds. Most breeds had data dating back to 1987: Brittany, English setter, Gordon setter, Norwegian Elkhound black and grey, and GSD; 1997: Eurasier and Portuguese Water dog; 2000: Danish - Swedish Farm dog.

### Breed selection

Based on a previous study of 14 dog breeds evaluated for the prevalence of LTV [2], nine breeds with more than 600 dogs were evaluated for LTV-status. The selection included a high LTV prevalence (> 20%): Brittany, Eurasier, Norwegian Elkhound black, and Portuguese Water dog. Additionally, the Norwegian Elkhound grey, closely related to the black variant, was included for comparison. Two setter breeds, the English setter and the Gordon setter, were selected for inter-breed comparisons. GSD was included in a comparison with previous studies on LTV heritability. Lastly, the Danish-Swedish Farm dog, a breed weighing < 10 kg, was included to represent a small-sized dogs.

### Inclusion criteria

Dogs were included in the study if they met the following criteria: hip radiographs and CHD grading according to the Fédération Cynologique Internationale (FCI) system [34]. Complete metadata, including a unique identity number, birth date, sex, and radiology date, were required [2]. According to CHD screening guidelines, dogs had to be aged ≥ 12 months at the time of radiography [2, 34].

### Classification of lumbosacral transitional vertebra (LTV)

LTV was classified into four types: LTV 0 represents normal lumbosacral anatomy; LTV 1 is characterised by an independent spinous process of the first sacral vertebra, separated from the medial sacral crest; LTV 2 is a symmetrical transitional vertebra separated from the sacrum by an intervertebral space; and LTV 3 exhibits asymmetrical morphology [20]. Radiographs were excluded if dense rectal faecal material, the os penis, or poor positioning obstructed relevant anatomy or if imaging quality was suboptimal due to artefacts, inadequate exposure, or distortion that hindered accurate classification. Additionally, radiographs were excluded if they lacked a visible last normal lumbar vertebra, which is essential as a reference for LTV classification. Two authors (JAB and CT) independently evaluated the radiographs and resolved any discrepancies through consensus. This information has been previously published [2].

### Classification of canine hip dysplasia (CHD)

The hip joint status of each dog was evaluated using the FCI five-grade system. This system classifies hips from A (normal joint) to E (severe CHD) based on the Norberg angle, degree of subluxation, acetabulum shape and depth, and secondary signs of osteoarthritis [34]. Only dogs with proper sedation, identification, left and right markings, and correct positioning were included [34]. This data was harvested from the NKK database [33].

### Estimation of pedigree-based heritability

LTV was recorded as a binary trait (unaffected/affected), which is justified because it seems that all phenotypes of LTV share a common genetic background [18]. CHD was recorded (A-E) as an ordered categorical trait (1–5) for the statistical analyses. Tables 1 and 2 show the number of records for LTV and CHD.

**Table 1:** The number of included dogs screened for canine hip dysplasia (CHD) by breed

Breed	CHD^1^	C%	D%	E%
Brittany	3813	27.7	9.8	0.6
Danish - Swedish Farm dog	2600	26.8	5.9	1.8
English setter	15,195	12.8	4.3	0.7
Eurasier	2241	11.8	4.7	1.9
German Shepherd dog	28,478	25.4	6.7	0.9
Gordon Setter	10,379	14.4	6.3	1.2
Norwegian Elkhound black	2572	11.0	4.1	0.7
Norwegian Elkhound grey	13,360	21.3	5.4	0.2
Portuguese Water dog	1875	19.1	4.8	0.8
Total number	80,513			

The table provides information on the number of dogs within each breed with a canine hip dysplasia (CHD) grading obtained from the Norwegian Kennel Club (NKK) database [33]. Most breeds had data dating back to 1987: Brittany, English Setter, Gordon Setter, Norwegian Elkhound black and grey, and GSD; 1997: Eurasier and Portuguese Water dog; 2000: Danish-Swedish Farm dog. CHD was graded according to the Fédération Cynologique Internationale (FCI) system, where A and B are considered free (not included), C indicates mild, D indicates moderate, and E indicates severe CHD [34]. The data are provided as percentages of dogs diagnosed with CHD grades C, D, and E

^1^ The total number of dogs within each dog breed included

**Table 2:** The number of dogs evaluated for lumbosacral transitional vertebra (LTV) by breed

Breed	LTV*	LTV**	LTV*** (%)
Brittany	1070	835	386
			(46.2)
Danish - Swedish Farm dog	1398	1027	149
			(14.5)
English setter	3521	2007	179
			(8.9)
Eurasier	1084	794	167
			(21.0)
German Shepherd dog	2843	1663	243
			(14.6)
Gordon setter	1371	1209	117
			(9.7)
Norwegian Elkhound black	692	679	195
			(28.6)
Norwegian Elkhound grey	1916	1361	242
			(17.8)
Portuguese Water dog	873	664	146
			(22.0)
Total number	14 768	10 239	1822
			(17.8)

The table provides information on the number of dogs within each breed evaluated for lumbosacral transitional vertebra (LTV) based on ventrodorsal radiographs. It includes the total number of dogs and the number of dogs with LTV types 1–3. Radiographic records from February 2014 to January 2022 were used. This period was chosen because radiographs were most likely available as digital pictures for a thorough classification of LTV [2]. LTV was classified as follows: LTV 0 represents normal lumbosacral anatomy; LTV 1 is characterised by an independent spinous process of the first sacral vertebra, separated from the medial sacral crest; LTV 2 is a symmetrical transitional vertebra separated from the sacrum by an intervertebral space; and LTV 3 exhibits asymmetrical morphology [20]

^*^ The total number of dogs evaluated within each breed, before exclusion; ^**^ The number of dogs within each breed included for evaluation of LTV; ^***^ The number of dogs within each breed affected with LTV types 1–3; %, percentage

The supplementary materials (Supplementary 1) provide complete pedigree data and details on model development. The tables present breed-specific statistics on pedigree information for dogs registered with LTV and CHD.

For each of the nine breeds, pedigree-based variance components were estimated in a bi-variate model using the following linear model:

y = Ryear + Sex + Ryear*Sex + litter + a + e.

Where: y = CHD or LTV, Ryear = Fixed effect of year of CHD recording, Sex = Fixed effect of sex, Ryear*Sex = Fixed interaction between Ryear and Sex, litter = Random effect of litter, a = Random additive genetic effect, e = Random residual error.

The assumed variance structure for the bi-variate model is shown in the supplementary material (Supplementary 2) [35].

Heritabilities were computed as: $\:{h}_{i}^{2}=\frac{{\sigma\:}_{{a}_{i}}^{2}}{{\sigma\:}_{{l}_{i}}^{2}+{\sigma\:}_{{a}_{i}}^{2}+{\sigma\:}_{{e}_{i}}^{2}}$, for *i =* CHD or LTV. The genetic correlation (r_g_) between LTV and CHD was computed as: $\:{r}_{g}=\frac{{\sigma\:}_{{a}_{LTV}{a}_{CHD}}}{\sqrt{{{\sigma\:}_{{a}_{LTV}}^{2}+\sigma\:}_{{a}_{CHD}}^{2}}}$. The asymptotic standard error of the estimated heritabilities and genetic correlations were computed using the AI matrix and a Taylor series approximation.

## Results

The heritability of LTV was low to moderate, ranging from 0.056 in the GSD to 0.314 in the Brittany, and was generally lower than for CHD (Table 3). Notably, the Norwegian Elkhound black (0.199 ± 0.087), Danish-Swedish Farm dog (0.124 ± 0.067), and English setter (0.121 ± 0.051) showed reasonable heritability, suggesting a stronger genetic component in these breeds. In contrast, the GSD had the lowest heritability (0.056 ± 0.051), indicating a weaker genetic contribution to LTV. The additive genetic variance for LTV was also low, ranging from 0.007 (GSD, Gordon setter) to 0.080 (Brittany). The Norwegian Elkhound black (0.042) and Eurasier (0.022) had relatively higher variance.

**Table 3:** Heritability and genetic variances for hip dysplasia, lumbosacral transitional vertebra and their correlation, with covariances

Breed	*h*^*2*^ CHD (SE)	Ad. gen. variance CHD^1^ (SE)	*h*^*2*^ LTV (SE)	Ad. gen. variance LTV^2^ (SE)	Gen. cor.^3^ (SE)	Cov.^4^ (SE)
Brittany	0.37	0.406	0.31	0.080	0.13	0.02
	(0.04)	(0.06)	(0.10)	(0.03)	(0.17)	(0.03)
Danish - Swedish Farm dog	0.52	0.551	0.12	0.016	-0.13	-0.01
	(0.48)	(0.07)	(0.07)	(0.01)	(0.20)	(0.02)
English setter	0.32	0.253	0.12	0.010	0.21	0.01
	(0.02)	(0.02)	(0.05)	(0.00)	(0.18)	(0.01)
Eurasier	0.29	0.267	0.14	0.022	-0.20	-0.02
	(0.06)	(0.06)	(0.07)	(0.01)	(0.26)	(0.02)
German Shepherd dog	0.25	0.367	0.06	0.007	0.06	0.00
	(0.0)	(0.02)	(0.05)	(0.01)	(0.05)	(0.02)
Gordon setter	0.36	0.355	0.08	0.007	0.38	0.02
	(0.03)	(0.03)	(0.06)	(0.01)	(0.26)	(0.01)
Norwegian Elkhound black	0.42	0.301	0.20	0.042	0.62	0.07
	(0.05)	(0.05)	(0.09)	(0.02)	(0.19)	(0.02)
Norwegian Elkhound grey	0.37	0.261	0.09	0.012	-0.05	0.00
	(0.02)	(0.02)	(0.05)	(0.01)	(0.20)	(0.01)
Portuguese Water dog	0.58	0.587	0.10	0.017	-0.05	-0.01
	(0.06)	(0.10)	(0.08)	(0.01)	(0.30)	(0.03)

The table provides information regarding the heritability (*h²*) of canine hip dysplasia (CHD) and lumbosacral transitional vertebra (LTV) for each dog breed, along with their corresponding additive genetic variances. It also includes the genetic correlation between LTV and CHD and the corresponding covariance

^1^ Additive genetic variance CHD; ^2^ Additive genetic variance LTV; ^3^ Genetic correlation LTV-CHD; ^4^ Additive genetic covariance

SE, standard error; Gen, genetic

Heritability for CHD varied from 0.254 (GSD) to 0.580 (Portuguese Water dog) (Table 3). The Portuguese Water dog (0.580 ± 0.058) and Danish-Swedish Farm dog (0.515 ± 0.048) had the highest values, reinforcing a strong genetic influence. The GSD (0.254 ± 0.015) and Eurasier (0.288 ± 0.057) had the lowest heritability, suggesting a greater environmental impact. The additive genetic variance for CHD followed a similar trend, highest in the Portuguese Water dog (0.587) and Danish-Swedish Farm dog (0.551) and lowest in the English setter (0.253) and Norwegian Elkhound grey (0.261).

Genetic correlations between LTV and CHD varied across breeds, with some showing positive associations (e.g., Norwegian Elkhound black: 0.615 ± 0.193) and others negative or near-zero correlations (e.g., Eurasier: -0.199 ± 0.255, Danish-Swedish Farm dog: -0.132 ± 0.195) (Table 3).

## Discussion

The first aim of this study was to estimate the heritability of LTV in nine dog breeds in Norway based on the same radiographs used for routine evaluation in the screening program for CHD. The heritabilities ranged from low to moderate, indicating that genetic improvements are possible by selective breeding strategies [9, 10]. The low heritability estimate for LTV in GSD is not consistent with previous studies where the heritability has been reported as moderate to high [8, 18, 19]. The prevalence of LTV in GSD in this study was also lower than previously reported [2, 4, 18]. The lower prevalence of LTV in Norwegian GSD is unexpected, given the presumed high exchange of breeding dogs between countries and the lack of selection for this trait in Norway. This variation may arise due to sampling differences, the classification system used, the statistical method employed for estimation, and the sample sizes [9, 10].

The second aim was to estimate the genetic correlation between LTV and CHD among the nine dog breeds. A positive genetic correlation would suggest that a breeding program targeting CHD could potentially reduce the prevalence of LTV. The results indicated breed differences, with both positive and negative correlations. This variability indicates that LTV and CHD do not share a consistent genetic basis across breeds, meaning one trait is not a reliable predictor of the other. Therefore, selection against one trait is unlikely to impact the other significantly, and both traits should be managed independently in breeding programs [36].

LTV type 2 and type 3 may have clinical implications related to CHD [2, 22, 23] and DLSS [24–26]. The clinical impact of CHD combined with lower back pain related to these LTV types remains unknown. However, these conditions - either independently or in combination - could have a negative impact on animal welfare and potentially lead to early retirement in working dogs [27–30].

A limitation of this study is that the pedigree-based LTV estimates for each breed are based on relatively small sample sizes. The low number of dogs in each of the original LTV classifications (Types 1–3) may reduce the statistical power of the model. To address this, we condensed these categories into a single “affected” group, increasing the number of observations per category and thereby enhancing statistical power. However, this binary classification, combined with reporting heritability on the observed rather than the underlying scale, may lead to a lower heritability estimate [19, 37]. Grouping LTV Types 1–3 as “affected” may be preferable, as they likely do not have a linear relationship and may share a common genetic background [18]. If LTV is to be included in systematic breeding programs, alternative models for estimating heritability should be explored to optimize and improve the precision of expected breeding progress estimates.

When the total population size is unknown, we cannot confirm that our data represents the whole population. However, we assume that most breeding dogs undergo hip dysplasia screening. Thus, our results are most likely valid for the breeding population, which forms the genetic basis for the next generation. We have no evidence to suggest that the prevalence of LTV differs between screened breeding dogs and unscreened dogs of the same breed.

This study relied exclusively on VD radiographs to classify LTV using a previously established classification system [20]. This system was chosen, as it is a straightforward system and its requirements are based on standard FCI radiographs for CHD screening, which was the only radiographic view available in the dataset.

Consequently, the terminology “lumbarisation” and “sacralisation” is not used [1]. It is important to note that the use of additional radiographic views or an alternative classification system for LTV could have yielded different results.

## Conclusions

This study demonstrates variation in the heritability of LTV and the genetic correlation with CHD in nine dog breeds in Norway, indicating that there is no consistent correlation between the two traits. LTV heritability is low to moderate, but genetic improvement would be feasible if breeders choose to put weight on the trait in the selection of breeding dogs. Given the small population size of the Norwegian Elkhound black, a primarily local breed with a small population size, routine evaluation and selection against CHD must be balanced with measures to reduce LTV prevalence and maintain genetic diversity. Overall, with accurate LTV grading, the heritability estimates indicate that it would be possible to reduce the prevalence of LTV using genetic selection if kennel clubs and dog breeders would sign up for such a program.

## Electronic supplementary material

Below is the link to the electronic supplementary material.

Supplementary Material 1

Supplementary Material 2

## Data Availability

The datasets used and/or analysed during the current study are available from the corresponding author on reasonable request.

## Declarations

### Prior publication

Data have not been published previously.

## References

[1] Morgan JP. Congenital anomalies of the vertebral column of the dog: a study of the incidence and significance based on a radiographic and morphologic study. Vet Radiol. 1968;9:21–9.

[2] Berg JA, Sævik BK, Lingaas F, Trangerud C. Lumbosacral transitional vertebra in 14 dog breeds in Norway: occurrence, risk factors and association with hip dysplasia. Vet J. 2024;303:106056. 10.1016/j.tvjl.2023.106056. 38092176 10.1016/j.tvjl.2023.106056

[3] Damur-Djuric N, Steffen F, Hässig M, Morgan JP, Flückiger MA. Lumbosacral transitional vertebrae in dogs: classification, prevalence, and association with sacroiliac morphology. Vet Radiol Ultrasound. 2006;47:32–8. 16429982 10.1111/j.1740-8261.2005.00102.x

[4] Fialová I, Paninárová M, Nečas A, Stehlík L, Proks P. Prevalence of lumbosacral transitional vertebrae in dogs in the Czech Republic. Acta Vet Brno. 2014;83:399–403.

[5] Kuricová M, Lipták T, Ledecký V. The occurence of lumbosacral transitional vertebra in two hunting breeds of dogs its association with hip dysplasia. Assiut Vet Med J. 2018;64:160–3.

[6] Lappalainen AK, Salomaa R, Junnila J, Snellman M, Laitinen-Vapaavuori O. Alternative classification and screening protocol for transitional lumbosacral vertebra in German shepherd dogs. Acta Vet Scand. 2012;54:27. 22549019 10.1186/1751-0147-54-27 PMC3403972

[7] Larsen JS. Lumbosacral transitional vertebrae in the dog. Vet Radiol. 1977;18:76–9.

[8] Wigger A, Julier-Franz C, Tellhelm B, Kramer M. Lumbosacral transitional vertebrae in the German shepherd dog: prevalence, classification, genetics, and association with canine hip dysplasia. Tierarztl Prax Ausg K Klientiere Heimtiere. 2009;37:7–13.

[9] Mackenzie S. Why heritability estimates differ: canine hip dysplasia. Canine Pract. 1985;12:19–22.

[10] Nicholas FW. Introduction to veterinary genetics. 3rd ed. Wiley-Blackwell; 2010.

[11] Fox SM, Burns J, Burt J. The dysplastic hip: a crippling problem in dogs. Vet Med. 1987;82:684–93.

[12] Leighton EA, Linn JM, Willham RL, Castleberry MW. A genetic study of canine hip dysplasia. Am J Vet Res. 1977;38:241–4. 842921

[13] King MD. Etiopathogenesis of canine hip dysplasia, prevalence, and genetics. Vet Clin North Am Small Anim Pract. 2017;4:753–67. 10.1016/j.cvsm.2017.03.001 28460694

[14] Swenson L, Audell L, Hedhammar Å. Prevalence and inheritance of and selection for hip dysplasia in seven breeds of dogs in Sweden and benefit: cost analysis of a screening and control program. J Am Vet Med Assoc. 1997;210:207–14. 9018354

[15] Soo M, Worth A. Canine hip dysplasia: phenotypic scoring and the role of estimated breeding value analysis. N Z Vet J. 2015;63:69–78. 25072401 10.1080/00480169.2014.949893

[16] Hedhammar Å. Swedish experiences from 60 years of screening and breeding programs for hip dysplasia—research, success, and challenges. Front Vet Sci. 2020;7:228. 10.3389/fvets.2020.00228. 32528980 10.3389/fvets.2020.00228 PMC7266929

[17] James HK, McDonnell F, Lewis TW. Effectiveness of canine hip dysplasia and elbow dysplasia improvement programs in six UK pedigree breeds. Front Vet Sci. 2020;6:490. 10.3389/fvets.2019.00490. 32010712 10.3389/fvets.2019.00490 PMC6974481

[18] Gluding D, Stock KF, Tellhelm B, Kramer M, Eley N. Genetic background of lumbosacral transitional vertebrae in German shepherd dogs. J Small Anim Pract. 2021;11:1–6. 10.1111/jsap.13380 34155659

[19] Ondreka N, Amort KH, Stock KF, Tellhelm B, Klumpp SW, Kramer M, et al. Skeletal morphology and morphometry of the lumbosacral junction in German shepherd dogs and an evaluation of the possible genetic basis for radiographic findings. Vet J. 2013;196:64–70. 22921082 10.1016/j.tvjl.2012.07.015

[20] Flückiger M, Geissbühler U, Lang J. Lumbosakrale Übergangswirbel: welche bedeutung Haben Sie für die gesundheit von Betroffenen Hunden? Schweiz Arch Tierheilkd. 2009;151:133–5. 19263383 10.1024/0036-7281.151.3.133

[21] Moeser CF, Wade CM. Relationship between transitional lumbosacral vertebrae and eight lumbar vertebrae in a breeding colony of Labrador retrievers and Labrador crosses. Aust Vet J. 2017;95:33–6. 28124426 10.1111/avj.12547

[22] Flückiger MA, Steffen F, Hässig M, Morgan JP. Asymmetrical lumbosacral transitional vertebrae in dogs May promote asymmetrical hip joint development. Vet Comp Orthop Traumatol. 2017;30:137–42. 28094414 10.3415/VCOT-16-05-0072

[23] Berg JA, Saevik BK, Lingaas F, Trangerud C. Evaluation of the effects of asymmetric lumbosacral transitional vertebra on pelvic morphology in dogs using ventrodorsal radiographs. Acta Vet Scand. 2025;67:4. 10.1186/s13028-024-00785-3. 39806492 10.1186/s13028-024-00785-3 PMC11727326

[24] Fluckiger MA, Damur-Djuric N, Hassig M, Morgan JP, Steffen F. A lumbosacral transitional vertebra in the dog predisposes to cauda equina syndrome. Vet Radiol Ultrasound. 2006;47:39–44. 16429983 10.1111/j.1740-8261.2005.00103.x

[25] Morgan JP, Bahr A, Franti CE, Bailey CS. Lumbosacral transitional vertebrae as a predisposing cause of cauda equina syndrome in German shepherd dogs: 161 cases (1987–1990). J Am Vet Med Assoc. 1993;202:1877–82. 8320160

[26] Berg JA, Saevik BK, Lingaas F, Trangerud C. Transitional lumbosacral vertebrae in black Norwegian elkhound and Brittany dogs: clinical findings and its association with degenerative lumbosacral stenosis. Acta Vet Scand. 2025;67:1–13. 39939978 10.1186/s13028-025-00797-7 PMC11816518

[27] Moore GE, Burkman KD, Carter MN, Peterson MR. Causes of death or reasons for euthanasia in military working dogs: 927 cases (1993–1996). J Am Vet Med Assoc. 2001;219:209–14. 11469577 10.2460/javma.2001.219.209

[28] Worth A, Sandford M, Gibson B, Stratton R, Erceg V, Bridges J, et al. Causes of loss or retirement from active duty for new Zealand Police German shepherd dogs. Anim Welf. 2013;22:167–74.

[29] Belshaw Z, Yeates J. Assessment of quality of life and chronic pain in dogs. Vet J. 2018;239:59–64. 30197111 10.1016/j.tvjl.2018.07.010

[30] Lindley S. The effects of pain on behaviour and behavioural problems part 3: aggression and compulsion. Companion Anim. 2012;17:50–4.

[31] Belshaw Z, Dean R, Asher L. Slower, shorter, sadder: a qualitative study exploring how dog walks change when the canine participant develops osteoarthritis. BMC Vet Res. 2020;16:1–8. 32156275 10.1186/s12917-020-02293-8 PMC7063782

[32] Christiansen SB, Kristensen AT, Sandøe P, Lassen J. Looking after chronically ill dogs: impacts on the caregiver’s life. Anthrozoos. 2013;26:519–33.

[33] Norwegian Kennel Club (NKK). https://www.dogweb.no/. Accessed Sep 11 2020.

[34] Fédération Cynologique Internationale. http://www.fci.be/en/. Accessed Apr 7 2022.

[35] Madsen P, Jensen J. A user’s guide to DUM. A package for analysing multivariate mixed models version. 2013;6:1–33.

[36] Hou Y, Wang Y, Lu X, Zhang X, Zhao Q. Monitoring hip and elbow dysplasia achieved modest genetic improvement of 74 dog breeds over 40 years in USA. PLoS ONE. 2013;8. doi.org/10.1371/. 10.1371/journal.pone.0076390 PMC3790730 24124555

[37] Grafen A, Hails R. Modern statistics for the life sciences. Oxford: Oxford University Press; 2010.

