# Supplementary material for: Genetic analyses of lumbosacral transitional vertebra and hip dysplasia in nine dog breeds in Norway
Source: Acta Vet Scand. 2025 May 26;67:25. doi: 10.1186/s13028-025-00810-z (PMC12107804; doi:10.1186/s13028-025-00810-z)
Supplement: Supplementary file 1 — Supplementary Material 1 [file 13028_2025_810_MOESM1_ESM.docx]

**LTV – Pedigree statistics-index**

The DMUTRACE program compute the amount of pedigree information for an individual as:

$$K_{Gen}=\sum_{i}^{Max\_gen} \frac{1}{2^{i}}\times{nk}_{i}$$

Where:

*Max_Gen* = maximum number of generations from an ancestor without data to a descendant with data

*i* = is generation back from the actual individuals

*nk_i_* = is the number of known ancestors in generation *i*

That is, each known parent contribute 1/2, each known grandparent 1/4, etc.

The pedigree completeness statistic by year of birth was computed as the average *K_gen_* for dogs with data (separately for LTV and CHD (not illustrated)).

1. **Pedigree completeness for dogs with LTV records;**

(The amount of pedigree information available (K_gen_) for the 9 breeds)

**Table 1:** Brittany

|  | K_gen_ | | | |
| --- | --- | --- | --- | --- |
| YOB | Mean | Min | Max | SD |
| 2013 | 5.16 | 2.00 | 6.47 | 1.14 |
| 2014 | 4.50 | 2.12 | 6.01 | 1.11 |
| 2015 | 4.93 | 2.00 | 7.03 | 1.17 |
| 2016 | 5.28 | 2.75 | 6.63 | 0.91 |
| 2017 | 5.03 | 1.00 | 6.71 | 1.20 |
| 2018 | 5.35 | 2.12 | 6.82 | 0.94 |
| 2019 | 5.17 | 2.00 | 6.54 | 1.08 |
| 2020 | 5.11 | 2.75 | 6.82 | 1.08 |
| Total | 5.12 | 1.00 | 7.03 | 1.09 |

**Table 2**: Danish Swedish Farm dog

|  | K_gen_ | | | |
| --- | --- | --- | --- | --- |
| YOB | Mean | Min | Max | SD |
| 2011 | 4.22 | 3.82 | 4.62 | 0.57 |
| 2012 | 4.46 | 4.46 | 4.46 | 0.59 |
| 2013 | 4.44 | 4.05 | 5.21 | 0.41 |
| 2014 | 4.32 | 3.79 | 4.88 | 0.49 |
| 2015 | 4.80 | 3.41 | 5.93 | 0.66 |
| 2016 | 5.07 | 2.84 | 6.83 | 0.70 |
| 2017 | 5.39 | 3.50 | 6.93 | 0.69 |
| 2018 | 5.39 | 3.33 | 6.98 | 0.73 |
| 2019 | 5.74 | 3.85 | 7.81 | 0.76 |
| 2020 | 5.86 | 3.81 | 7.11 | 0.56 |
| Total | 5.42 | 2.84 | 7.81 | 0.76 |

**Table 3:** English setter

|  | K_gen_ | | | |
| --- | --- | --- | --- | --- |
| YOB | Mean | Min | Max | SD |
| 2011 | 6.50 | 5.61 | 7.13 | 0.68 |
| 2012 | 6.24 | 4.48 | 7.49 | 1.11 |
| 2013 | 6.05 | 4.12 | 7.27 | 0.80 |
| 2014 | 6.17 | 2.00 | 7.70 | 1.21 |
| 2015 | 6.44 | 2.31 | 8.06 | 1.17 |
| 2016 | 6.44 | 2.00 | 8.02 | 1.10 |
| 2017 | 6.67 | 2.50 | 8.28 | 1.03 |
| 2018 | 6.73 | 2.00 | 8.43 | 1.03 |
| 2019 | 6.76 | 2.00 | 8.46 | 0.97 |
| 2020 | 7.01 | 3.53 | 8.58 | 1.19 |
| 2021 | 8.27 | 7.96 | 8.58 | 0.44 |
| Total | 6.62 | 2.00 | 8.58 | 1.09 |

**Table 4:** Eurasier

|  | K_gen_ | | | |
| --- | --- | --- | --- | --- |
| YOB | Mean | Min | Max | SD |
| 2013 | 3.30 | 2.00 | 4.14 | 1.14 |
| 2014 | 4.21 | 3.25 | 5.27 | 0.59 |
| 2015 | 4.53 | 2.44 | 5.53 | 0.69 |
| 2016 | 4.74 | 2.62 | 5.83 | 0.61 |
| 2017 | 4.64 | 2.00 | 6.08 | 0.84 |
| 2018 | 5.06 | 3.49 | 6.23 | 0.62 |
| 2019 | 4.98 | 3.00 | 6.49 | 0.71 |
| 2020 | 4.96 | 2.00 | 6.13 | 0.68 |
| 2021 | 3.96 | 3.22 | 5.43 | 1.27 |
| Total | 4.81 | 2.00 | 6.49 | 0.73 |

**Table 5:** Gordon setter

|  | K_gen_ | | | |
| --- | --- | --- | --- | --- |
| YOB | Mean | Min | Max | SD |
| 2011 | 5.31 | 4.71 | 5.92 | 0.86 |
| 2013 | 6.28 | 3.46 | 7.35 | 0.82 |
| 2014 | 6.43 | 2.00 | 8.12 | 1.17 |
| 2015 | 6.59 | 3.02 | 7.99 | 1.09 |
| 2016 | 6.76 | 2.12 | 8.46 | 1.20 |
| 2017 | 7.10 | 2.00 | 8.64 | 1.25 |
| 2018 | 6.92 | 2.25 | 8.69 | 1.35 |
| 2019 | 7.18 | 2.12 | 8.96 | 1.30 |
| 2020 | 7.48 | 2.31 | 8.85 | 1.03 |
| Total | 6.93 | 2.00 | 8.96 | 1.25 |

**Table 6:** Norwegian Elkhound grey

|  | K_gen_ | | | |
| --- | --- | --- | --- | --- |
| YOB | Mean | Min | Max | SD |
| 2011 | 6.83 | 3.77 | 7.76 | 0.89 |
| 2012 | 7.08 | 4.81 | 8.50 | 0.96 |
| 2013 | 7.24 | 5.58 | 8.43 | 0.75 |
| 2014 | 7.52 | 4.64 | 8.78 | 0.88 |
| 2015 | 7.53 | 5.14 | 9.09 | 0.83 |
| 2016 | 7.83 | 5.56 | 9.22 | 0.72 |
| 2017 | 7.80 | 5.82 | 9.22 | 0.69 |
| 2018 | 7.91 | 5.93 | 9.61 | 0.84 |
| 2019 | 8.32 | 4.84 | 9.67 | 0.85 |
| 2020 | 8.37 | 6.82 | 9.73 | 0.53 |
| Total | 7.84 | 3.77 | 9.73 | 0.85 |

**Table 7:** Norwegian Elkhound black

|  | K_gen_ | | | |
| --- | --- | --- | --- | --- |
| YOB | Mean | Min | Max | SD |
| 2013 | 7.90 | 7.13 | 8.59 | 0.44 |
| 2014 | 8.33 | 7.09 | 9.21 | 0.51 |
| 2015 | 8.33 | 7.53 | 9.77 | 0.38 |
| 2016 | 8.57 | 7.71 | 9.43 | 0.46 |
| 2017 | 8.82 | 7.56 | 9.62 | 0.42 |
| 2018 | 8.98 | 8.21 | 9.73 | 0.36 |
| 2019 | 9.24 | 8.57 | 9.96 | 0.29 |
| 2020 | 9.56 | 8.25 | 10.35 | 0.45 |
| Total | 8.87 | 7.09 | 10.35 | 0.60 |

Table 8: Portuguese Water dog

|  | K_gen_ | | | |
| --- | --- | --- | --- | --- |
| YOB | Mean | Min | Max | SD |
| 2011 | 5.02 | 5.02 | 5.02 |  |
| 2014 | 3.95 | 3.22 | 4.63 | 0.65 |
| 2015 | 4.72 | 2.50 | 6.11 | 0.84 |
| 2016 | 4.88 | 2.69 | 6.36 | 0.69 |
| 2017 | 5.23 | 2.69 | 6.38 | 0.80 |
| 2018 | 4.86 | 2.75 | 6.57 | 0.95 |
| 2019 | 5.52 | 2.12 | 6.54 | 0.87 |
| 2020 | 5.82 | 4.39 | 6.67 | 0.58 |
| 2021 | 6.45 | 6.45 | 6.45 |  |
| Total | 5.11 | 2.12 | 6.67 | 0.89 |

**Table 9:** German Shepherd dog

|  | K_gen_ | | | |
| --- | --- | --- | --- | --- |
| YOB | Mean | Min | Max | SD |
| 2011 | 5.27 | 5.27 | 5.27 |  |
| 2014 | 6.43 | 3.55 | 7.88 | 1.35 |
| 2015 | 6.25 | 3.29 | 8.13 | 1.66 |
| 2016 | 5.71 | 2.12 | 8.63 | 1.57 |
| 2017 | 6.08 | 1.67 | 8.92 | 1.69 |
| 2018 | 6.17 | 2.25 | 8.47 | 1.57 |
| 2019 | 6.15 | 1.80 | 9.07 | 1.68 |
| 2020 | 6.22 | 2.12 | 8.65 | 1.74 |
| 2021 | 7.53 | 5.77 | 8.53 | 1.21 |
| Total | 6.07 | 1.67 | 9.07 | 1.65 |
