# Supplementary material for: Genetic analyses of lumbosacral transitional vertebra and hip dysplasia in nine dog breeds in Norway
Source: Acta Vet Scand. 2025 May 26;67:25. doi: 10.1186/s13028-025-00810-z (PMC12107804; doi:10.1186/s13028-025-00810-z)
Supplement: Supplementary file 2 — Supplementary Material 2 [file 13028_2025_810_MOESM2_ESM.docx]

The assumed variance structure for the bi-variate model is:

$$Var\left[ \begin{matrix} l \\ a \\ e \end{matrix} \right]=\left[ \begin{matrix} L\otimes I_{l} & 0 & 0 \\ 0 & G\otimes A & 0 \\ 0 & 0 & R\otimes I_{e} \end{matrix} \right]$$

Where *A* is the additive genetic relationship matrix, *I_l_* and *I_e_* are identity matrices of appropriate size, ⊗ is the Kronecker product, L, G, and R are the litter, genetic and residual variance-covariance matrices, given by:

$L=\left[ \begin{matrix} \sigma_{l_{LTV}}^{2} & \sigma_{l_{LTV}l_{CHD}} \\ \sigma_{l_{LTV}l_{CHD}} & \sigma_{l_{2}}^{2} \end{matrix} \right]$, $G=\left[ \begin{matrix} \sigma_{a_{LTV}}^{2} & \sigma_{a_{LTV}a_{CHD}} \\ \sigma_{a_{LTV}a_{CHD}} & \sigma_{a_{2}}^{2} \end{matrix} \right]$, $R=\left[ \begin{matrix} \sigma_{e_{LTV}}^{2} & \sigma_{e_{LTV}e_{CHD}} \\ \sigma_{e_{LTV}e_{CHD}} & \sigma_{e_{2}}^{2} \end{matrix} \right]$The within-breed variance components were estimated using the Average Information Restricted Maximum Likelihood (AI-REML) module from the DMU package [35].
